# Stable Low-Voltage Organic Memristors Enabled by Templated Crystallization and Quantum-Dot-Regulated Filament Formation

**DOI:** 10.3390/ma19143029

**Published:** 2026-07-14

**Authors:** Qi Lei, Yonghua Tu, Zilong Yan, Junqing Wei, Boning Han, Haiwei Zhang, Yangyang Xie, Kailiang Zhang

**Affiliations:** 1 Tianjin Key Laboratory of Film Electronic and Communication Devices, School of Integrated Circuit Science and Engineering, Tianjin University of Technology, Tianjin 300384, China; lq1150506149@163.com (Q.L.); a1944213349@163.com (Y.T.); 18303365869@163.com (Z.Y.); zhanghaiwei@email.tjut.edu.cn (H.Z.); kailiang_zhang2007@163.com (K.Z.); 2 Tianjin Key Laboratory for Photoelectric Materials & Devices, School of Materials Science and Engineering, Tianjin University of Technology, Tianjin 300384, China; 69023591@email.tjut.edu.cn

**Keywords:** organic memristor, TIPS-pentacene, quantum dots, crystallization engineering, conductive filament regulation

## Abstract

Organic memristors are attractive building blocks for neuromorphic computing owing to their intrinsic synaptic functionalities and solution-processability. However, their operational instability remains a major challenge, primarily arising from poorly controlled semiconductor crystallization and stochastic conductive filament formation. Here, we report a high-performance solution-processed organic memristor based on a TIPS-pentacene/PMMA/CdSe-ZnS quantum-dot hybrid system, in which a dual-engineering strategy is employed to simultaneously regulate film crystallization and filament dynamics. Specifically, the PMMA matrix templates the molecular ordering of TIPS-pentacene to improve film uniformity and crystallinity, while CdSe/ZnS quantum dots locally modulate the electric field to direct and confine conductive filament formation. As a result, the device exhibits ultralow and highly uniform switching voltages (0.473 V for set and −0.430 V for reset), suppressed device-to-device variation, long retention exceeding 10^4^ s, and endurance over 1200 switching cycles. In addition, the memristor supports multilevel data storage and successfully emulates key synaptic functions, including long-term potentiation/depression, paired-pulse facilitation, and spike-timing-dependent plasticity. This work provides a materials-level strategy for achieving reliable and low-power organic memristors, offering a viable route toward high-density nonvolatile memory and neuromorphic computing hardware.

## 1. Introduction

Memristors have emerged as key hardware elements for next-generation neuro-morphic computing because they can integrate information storage, signal processing, and sensory functionalities within compact and energy-efficient architectures [1,2,3,4]. Compared with their inorganic counterparts, organic memristors offer several distinctive advantages, including low-cost solution processability, mechanical flexibility, and compatibility with large-area and unconventional substrates [5,6]. These attributes make them particularly attractive for flexible intelligent electronics and bioinspired computing systems. However, despite these merits, the practical deployment of organic memristors remains severely constrained by insufficient operational stability and poor switching reproducibility [7]. A central challenge originates from the intrinsic structural disorder of solution-processed organic semiconductors. During film formation, rapid solvent evaporation and uncontrolled molecular self-assembly often produce discontinuous microstructures, nonuniform crystallization, and abundant trap-rich grain boundaries [8,9,10]. Such morphological and structural inhomogeneities lead to highly stochastic conductive filament (CF) nucleation and rupture, resulting in broad distributions of switching voltages, large cycle-to-cycle and device-to-device variations, and limited endurance [11,12,13]. Therefore, achieving deterministic resistive switching in organic memristors requires the simultaneous regulation of both semiconductor crystallization and filament evolution.

Considerable efforts have been devoted to improving the uniformity of resistive switching through interface engineering, additive incorporation, and nanostructure-assisted field modulation. Among these strategies, quantum dots (QDs) have attracted particular attention because they can locally concentrate the electric field, regulate charge trapping, and direct ion migration at the nanoscale [14,15,16]. For example, self-assembled PbS QDs have been shown to improve switching uniformity in inorganic memristors by guiding filament growth, while CdSe/CdS and carbon QDs have been introduced into TaOx- and Ag-based switching systems to reduce operating voltage and enhance endurance [17,18,19]. Despite these encouraging advances, the use of QDs in organic memristors remains largely unexplored. More importantly, QD-mediated filament control alone is unlikely to fully resolve switching randomness if the organic semiconductor matrix itself still suffers from poor crystallinity and film discontinuity. Thus, a more effective approach should combine structural ordering of the organic film with nanoscale control over filament formation.

In polymer-assisted organic memristors, polymers are mainly employed to improve film-forming quality, suppress leakage currents, or tune phase separation, but they generally do not provide nanoscale guidance for conductive filament formation. In contrast, QD-assisted memristors mainly rely on local electric-field enhancement or charge-trapping effects to regulate filament growth, while the crystallinity and continuity of the organic semiconductor matrix are usually not simultaneously optimized. Therefore, a rational dual-engineering strategy should integrate matrix-level crystallization regulation with nanoscale filament modulation. To implement this concept, an organic semiconductor whose electrical performance is strongly governed by film morphology and molecular ordering is highly desirable.

TIPS-pentacene is a representative small-molecule organic semiconductor with high charge-transport capability and excellent solution-processability, making it an attractive active material for organic electronic devices. However, its film formation is highly sensitive to solvent evaporation kinetics and substrate wetting behavior, often resulting in poor coverage and uncontrolled crystal growth under conventional spin-coating conditions. Polymer-assisted crystallization provides a promising route to overcome this limitation. In particular, an insulating polymer matrix can promote film continuity, regulate molecular assembly, and improve morphological uniformity without fundamentally compromising the semiconducting functionality of the active phase. If combined with appropriately dispersed nanostructured field regulators, such a hybrid design may offer a viable pathway toward stable and low-voltage organic memristors.

Here, we report a solution-processed ternary hybrid organic memristor based on TIPS-pentacene, poly(methyl methacrylate) (PMMA), and CdSe/ZnS QDs. The device is constructed through a dual-engineering strategy that simultaneously targets the two major sources of switching instability in organic memristors. Specifically, PMMA serves as a crystallization template to promote the ordered assembly and continuous film formation of TIPS-pentacene, while CdSe/ZnS QDs act as nanoscale electric-field modulators that guide and spatially confine conductive filament formation. Benefiting from this synergistic design, the optimized device exhibits ultralow and highly uniform switching voltages (0.473 V for set and −0.430 V for reset), low cycle-to-cycle and device-to-device variation, retention exceeding 10^4^ s, and endurance over 1200 switching cycles. In addition, the device supports multilevel resistance states and reliably emulates essential synaptic functions, including long-term potentiation/depression (LTP/LTD), paired-pulse facilitation (PPF), and spike-timing-dependent plasticity (STDP). This work establishes a materials-engineering strategy for achieving deterministic switching in solution-processed organic memristors and advances their prospects for neuromorphic computing and high-density nonvolatile memory applications.

## 2. Experimental Section

### 2.1. Materials

Sulfur powder (Sulfur, S, 98.5%), 2-octadecene (1-octadecene, ODE, 90%), stearic acid (98%), and trioctylphosphine (TOP, 85%) were purchased from TCI (Shanghai) Chemical Industry Development Co., Ltd. (Shanghai, China). Selenium powder (selenium powder, Se, 325 mesh, 99.5%) was purchased from Alfa Aesar (China) Chemical Co., Ltd. (Shanghai, China). Cadmium stearate (Cd(St)_2_) was purchased from Shanghai Dibo Chemical Technology Co., Ltd. (Shanghai, China). Zinc acetate (Zn(Ac)_2_, 99.5%) and 6,13-bis(triisopropylsilylethynyl)pentacene (TIPS, RG, 97%) were purchased from Shanghai Titan Technology Co., Ltd. (Shanghai, China). Ethanol (99.7%, analytical grade) and dimethylbenzene (xylene, analytical grade) were purchased from Tianjin Damao Chemical Reagent Co., Ltd. (Tianjin, China). Chlorobenzene (AR, 99%) was purchased from Jiangtian Chemical Industry, Tianjin, China. Poly(methyl methacrylate) was purchased from Chongqing Platinum Strontium Titanium Technology Co., Ltd. (Chongqing, China). All other chemicals were of analytical grade and used without further purification unless otherwise stated.

### 2.2. Synthesis of CdSe/ZnS QDs

Put 2 mmol of cadmium stearate and 0.2 mmol of stearic acid into a 50 mL three-necked flask. Add 10 mL of octene, stir thoroughly, and perform nitrogen bubbling (alternating nitrogen and vacuuming) to remove the air from the flask (the main purpose is to remove oxygen), then continue stirring and maintaining the nitrogen environment. Heat the three-necked flask to 300 °C using the temperature controller and heating jacket. Next, rapidly inject 0.5 mL of TOP-Se solution (2 mmol of Se powder dissolved in 1 mL of TOP, stirred thoroughly to dissolve) into the three-necked flask using a syringe. Heat at 300 °C for 2 min. Then, rapidly inject 0.5 mL of TOP-S solution (4 mmol of S powder dissolved in 2 mL of TOP, stirred thoroughly to dissolve) into the three-necked flask. Maintain the temperature at 300 °C for 40 min, then allow the flask to cool to room temperature. Afterward, add 1 mmol of cadmium stearate, 2 mmol of zinc acetate, and 5 mL of ODE to the three-necked flask, and stir thoroughly. After sufficient nitrogen bubbling, while maintaining stirring and nitrogen atmosphere, heat the flask to 160 °C. At this point, slowly inject 1.5 mL of TOP-S (prepared as previously described) into the three-necked flask and continue heating at 160 °C for 4 h. Finally, stop heating and allow the flask to cool to room temperature. Transfer the obtained solution into a centrifuge tube, add an appropriate amount of ethanol, mix thoroughly, and then centrifuge to collect QDs from the precipitate. Subsequently, dissolve the precipitate in an appropriate amount of xylene, add more ethanol, and centrifuge again to clean. This will yield the desired quantum dot precipitate.

### 2.3. Device Fabrication and Characterization

Chlorobenzene was used as the solvent to prepare a 2 mg/mL PMMA solution, into which 10 mg of TIPS-pentacene was dissolved. Magnetic stirring was carried out at 500 rpm and 45 °C for 10 h to obtain a uniform precursor solution. Subsequently, 5 mg, 10 mg, and 20 mg of cleaned CdSe/ZnS QD precipitates were added to the 10 mg/mL TIPS-Pentacene solution to prepare mixed solutions with mass ratios of 1:2, 1:1, and 2:1. All mixed solutions were stirred under the same conditions (500 rpm, 45 °C) for 10 h. After stirring, they were filtered through a 0.22 μm molecular sieve, and the filtrate was collected for future use. Four FTO substrates, cleaned with ethanol, glass cleaner, and ultrasonic treatment, followed by ozone treatment and cleaning with a plasma cleaner, were selected. A total of 50 μL of mixed solutions, both without QDs and containing QDs in different proportions, were dropped onto the surface of the FTO substrates. Spin-coating was performed at 3000 rpm for 30 s. The films were annealed at 90 °C for 1 h after spin-coating was completed. Finally, Ag electrodes were evaporated onto the composite films using shadow masks to complete the device fabrication.

### 2.4. Electrical Measurements

All electrical measurements are carried out by using a semiconductor parameter analyzer (Keysight Agilent B1500A, Agilent Technologies, Santa Rosa, CA, USA). A voltage signal is applied to the Ag top electrode, while the FTO bottom electrode is grounded.

### 2.5. Material Characterizations

The materials are characterized by X-ray photoelectron spectroscopy (Escalab 250Xi, Thermo Fisher Scientific, Waltham, MA, USA), a Raman spectrometer (INVIA0618-02, Renishaw, Gloucestershire, UK), a high-resolution transmission electron microscope (PEI Talos F200X, Thermo Fisher Scientific, Waltham, MA, USA), a scanning electron microscope (Zeiss Merlin Compact, Carl Zeiss Microscopy GmbH, Jena, Germany), an optical microscope (Olympus, M1T1705678, Tokyo, Japan), and an X-ray diffractometer (Rigaku Smartlab 9 kW, Rigaku Corporation, Tokyo, Japan).

## 3. Results and Discussion

**Figure 1:**
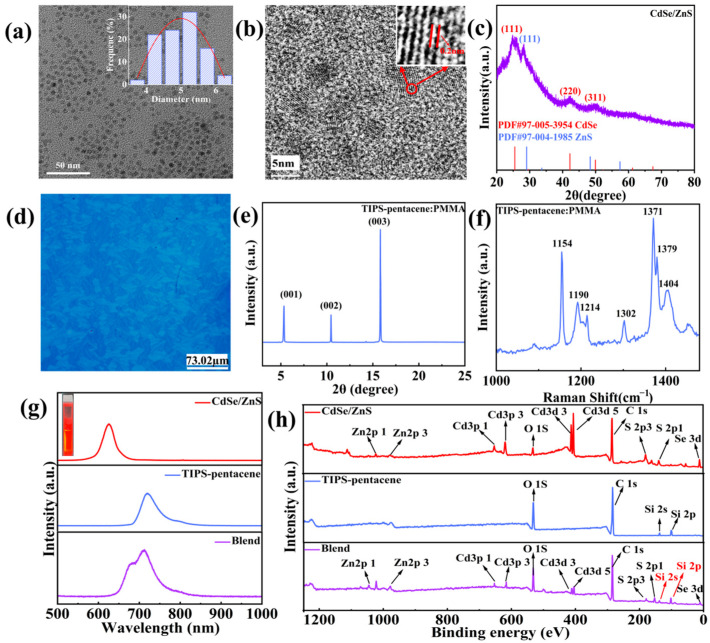
(**a**) TEM image and particle size distribution fitting curve of CdSe/ZnS QDs. (**b**) HRTEM image and lattice spacing of CdSe/ZnS QDs. (**c**) XRD patterns of CdSe/ZnS QDs. (**d**) Optical microscope image of a TIPS-pentacene:PMMA blend film spin-coating from a solution with a PMMA concentration of 2 mg/mL. (**e**) XRD patterns of TIPS-pentacene. (**f**) Raman spectra of TIPS-pentacene. (**g**) PL spectra of QDs, TIPS-pentacene, and their blend under 365 nm light excitation. (**h**) XPS full survey spectra of CdSe/ZnS QDs, TIPS-pentacene, and the blend solution.

The fabrication of the PMMA-QD hybrid memristor is schematically illustrated in Figure S1, in which the QDs adopt a CdSe/ZnS core–shell structure. As shown in Figure 1a, TEM imaging revealed well-dispersed QDs with uniform morphology and a narrow size distribution centered around 5 nm. HRTEM imaging (Figure 1b) further resolved a lattice spacing of 0.2 nm, while XRD spectra (Figure 1c) displayed distinctive diffraction peaks at 2θ = 24.8°, 42.2°, and 50.3°, corresponding to the (111), (220), and (311) planes of the cubic CdSe phase, along with a ZnS-associated peak at 2θ = 28°, collectively verifying the core–shell structure of the QDs [20,21].

To address the inherent film-forming challenges of TIPS-pentacene, we introduced a PMMA polymer matrix to template the crystallization process. Initial attempts to deposit pristine TIPS-pentacene from chlorobenzene solutions (2–10 mg/mL) resulted in discontinuous films with poor substrate coverage and no observable crystalline domains (Figure S2a–c), leading to no resistive switching (RS) behavior and frequent device failure (Figure S2d–f). In contrast, the incorporation of PMMA at an optimal concentration of 2 mg/mL enabled the formation of a continuous, highly crystalline film (Figure 1d and Figure S3). This morphological improvement was corroborated by XRD analysis (Figure 1e), where distinct diffraction peaks at 2θ = 5.96°, 10.74°, and 16.54° confirmed the development of a well-ordered α-phase crystalline structure of TIPS-pentacene [22,23]. Raman spectroscopy (Figure 1f) further affirmed the structural integrity, showing characteristic vibrational modes at 1154, 1178, 1190, 1214, 1302, 1353, and 1373 cm^−1^ [24,25]. It is noteworthy that deviations from the optimal PMMA concentration led to degraded film uniformity (Figure S4a,b) and erratic RS behavior (Figure S4c,d), underscoring the critical role of the polymer matrix in attaining a balance between structural order and electronic functionality.

Leveraging the PMMA matrix as a crystallization template, we achieved the synergistic integration of TIPS-pentacene with CdSe/ZnS QDs, yielding a high-quality hybrid film that serves as the material cornerstone for the high-performance memristors demonstrated in this work. Energy-dispersive X-ray spectroscopy (EDS) elemental mapping of the composite film reveals that the QD constituents are uniformly dispersed throughout the TIPS-pentacene/PMMA matrix, including C, O, Si, Cd, Se, Zn, and S elements (Figure S5). Photoluminescence (PL) spectra of the ternary hybrid (Figure 1g) exhibited a fused emission profile with peaks at 680 nm and 710 nm, situated between the intrinsic emissions of the QDs (625 nm red emitting) and TIPS-pentacene (720 nm), indicating electronic interaction between the components. XPS survey spectra (Figure 1h) confirmed the coexistence of elemental signatures from both QDs (Zn 2p, Cd 3d, Se 3d, S 2p) and TIPS-pentacene (Si 2s, Si 2p, C 1s, O 1s), suggesting homogeneous blending and interfacial compatibility. Additionally, UV-Vis absorption spectra (Figure S6) showed that the hybrid film retained the characteristic absorption of CdSe/ZnS QDs at 610 nm and the broad π-π* transition features of TIPS-pentacene between 350 and 700 nm, confirming the preserved optoelectronic properties of both components within the composite. Cross-sectional SEM imaging (Figure S7) verified the well-defined sandwich architecture of the final memristor device and the active layer was ~44 nm.

**Figure 2:**
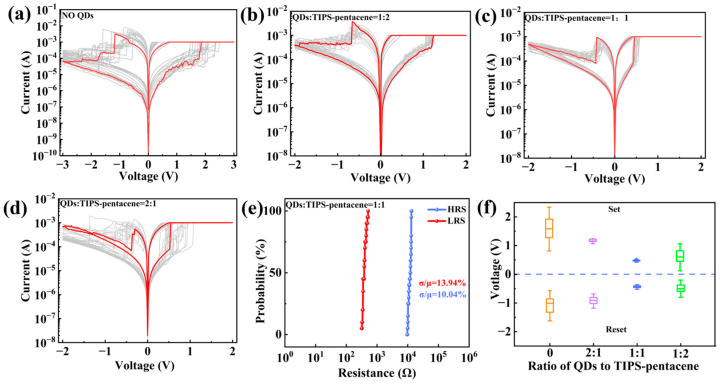
I–V characteristics and statistical analysis of memristors with different QDs:TIPS-pentacene ratios. (**a**) I–V curve of the TIPS-pentacene memristor without QDs. (**b**) I–V curve of the memristor with a 1:2 QDs:TIPS-pentacene ratio. (**c**) I–V curve of the memristor with a 1:1 ratio. (**d**) I–V curve of the memristor with a 2:1 ratio. (**e**) Cumulative probability distribution of the HRS/LRS for the memristor at a 1:1 ratio. (**f**) Box plot of the set/reset threshold voltages of the memristors under different ratio conditions.

To quantitatively assess the role of CdSe/ZnS QDs in optimizing memristive performance, we systematically investigated devices with varying QDs/TIPS-pentacene ratios. Cyclic I–V measurements (Figure 2a–d) reveal a pronounced dependency of switching characteristics on QDs content. The QDs-free control device (Figure 2a) exhibits the highest and most erratic switching voltages with poor LRS/HRS stability. Introducing a small QDs fraction (Figure 2b) immediately reduces operational voltages and improves uniformity, attributable to the localized field-enhancement effect of QDs that guides more deterministic CF formation. As demonstrated in recent simulation studies, high-dielectric constant QDs act as nanoscale capacitive centers that concentrate electric fields at their poles [17,26]. This intensified local field not only lowers the kinetic barrier for ionic migration but also creates preferential pathways for conductive charge carriers, thereby guiding more deterministic and vertically aligned CF formation compared to the stochastic growth observed in pristine films [27,28]. The optimal performance is achieved at a 1:1 QD-to-TIPS-pentacene ratio (Figure 2c), yielding the most uniform I–V characteristics and robust LRS/HRS states, indicative of stable and well-defined CFs. This specific ratio likely represents an ideal balance where the inter-QD spacing is minimized sufficiently to maintain a continuous, guided conduction front without inducing significant electrostatic interference between adjacent dots. Further increasing the QD content (Figure 2d) degrades performance, as excessive QDs likely promote multiple, competing filamentary paths due to overlapping high-field zones, which disrupt the directional control of CF growth and lead to chaotic switching events [27].

The optimal performance is achieved at a 1:1 QDs-to-TIPS-pentacene ratio (Figure 2c), yielding the most uniform I–V characteristics and robust LRS/HRS states, indicative of stable and well-defined CFs. Further increasing the QD content (Figure 2d) degrades performance, as excessive QDs likely promote multiple, competing filamentary paths. Quantitative analysis of resistance state fluctuations via the coefficient of variation (CV) confirms this trend (Figure 2e). The 1:1 device demonstrates superior consistency with LRS and HRS CVs of 10.04% and 13.94%, respectively, significantly lower than the QDs-free (38.68%/45.13%) and high-QDs content (29.01%/18.52%) devices, as shown in Figure S8. Statistical analysis of threshold voltages further corroborates the optimized performance at the 1:1 ratio (Figure 2f). This device exhibits ultralow, uniform average switching voltages of (0.473 V/−0.430 V) with minimal variability (CVs of 5.1%/10.7%). In contrast, the QDs-free device operates at higher voltages (1.646 V/−1.078 V) with substantial variability (CVs 22.1%/29.5%). These results unequivocally verify that an optimal QD incorporation is critical for minimizing operational power and enhancing switching reproducibility, as the QD-mediated field confinement effectively homogenizes the nucleation sites and growth trajectories of CFs, transitioning the switching mechanism from erratic, bulk-dominated growth to a regulated, interface-controlled process [26,27,28].

**Figure 3:**
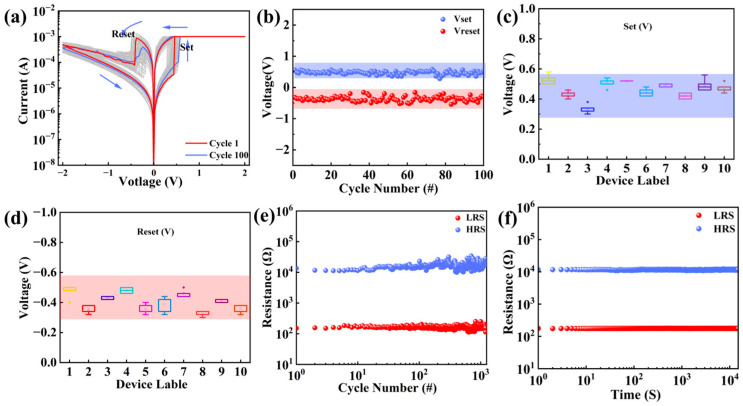
(**a**) I–V curves of the memristor over 100 cycles with a ratio of 1:1. (**b**) Statistical distribution of set/reset threshold voltages over 100 cycles for the memristor with a QDs:TIPS-pentacene ratio of 1:1. (**c**) Box plot of set voltage from 10 devices. (**d**) Box plot of reset voltage from 10 devices. (**e**) Endurance test of memristors with a QDs:TIPS-pentacene ratio of 1:1. (**f**) Retention test of memristors with a QDs:TIPS-pentacene ratio of 1:1.

The optimized 1:1 QDs:TIPS-pentacene memristor exhibits outstanding operational reliability, a critical metric for practical applications. As shown in Figure 3a, 100 consecutive I–V cycles under ±2 V sweeping reveal highly concentrated set and reset voltages, distributed narrowly between 0.3 to 0.5 V and −0.2 to −0.5 V, respectively. Statistical analysis further confirms the stability of these parameters over continuous operation, with average set/reset voltages of 0.468 V and −0.42 V and corresponding CVs of 10.9% and 15.6% (Figure 3b). Excellent device-to-device reproducibility was also verified across ten independently fabricated units (Figure 3c,d), with switching voltages consistently centered around 0.4 V/−0.4 V and minimal inter-device fluctuation. Although minor variations in the I–V window were observed (Figure S9), the overall uniformity underscores the robustness of our hybrid film fabrication process, with potential further improvements possible through more precise control of QD dispersion. Most notably, the device demonstrates remarkable endurance, maintaining clear LRS/HRS differentiation over 1200 consecutive switching cycles (Figure 3e). Retention capabilities exceed 10^4^ s under a 0.1 V read voltage (Figure 3f), significantly outperforming devices with non-optimal QD ratios (Figure S10). These results collectively affirm that the PMMA-QD hybrid structure not only enhances switching uniformity but also ensures long-term functional integrity, positioning it as a highly promising candidate for reliable neuromorphic hardware.

**Figure 4:**
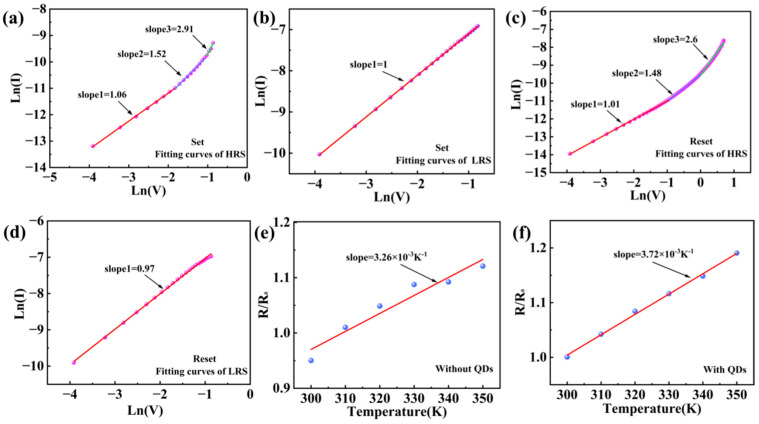
(**a**) Fitted curve of the memristor’s HRS with QDs. (**b**) Fitted curve of the memristor’s LRS with QDs. (**c**) Fitted curve of the memristor’s HRS without QDs. (**d**) Fitted curve of the memristor’s LRS without QDs. (**e**) Temperature-dependent curve of the memristor’s LRS without QDs. (**f**) Temperature-dependent curve of the memristor’s LRS with QDs.

To unravel the conductive mechanism, we performed detailed electrical and thermal analysis. The I–V characteristics plotted on a logarithmic scale (Figure 4a–d) for both QDs-based and QDs-free devices conform to the space-charge-limited-current (SCLC) model in the HRS or LRS. For the QDs-based device (Figure 4a), the HRS fitting yields slopes of 1.06 (Ohmic region, I∝V), 1.79 (Child’s law region, I∝V^2^), and 3.67 (trap-filled limit region), while the LRS shows a single Ohmic slope of 1.06 (Figure 4b), confirming the formation of metallic CFs.

Further insight was gained through temperature-dependent measurements. The resistance in the LRS decreased with increasing temperature (300–350 K) for both devices, exhibiting metallic behavior. Linear fitting to Equation (1):

R(T) = R0[1 + α(T − T0)](1)

revealed a higher temperature coefficient of resistance (TCR) for the QD-based device (3.72 × 10^−3^ K^−1^) compared to the QD-free device (3.26 × 10^−3^ K^−1^). The value for the QD-incorporated device is closer to that of defect-free single-crystal Ag nanowires (4 × 10^−3^ K^−1^), indicating the formation of more robust and crystalline Ag CFs (Figure 4e,f).

**Figure 5:**
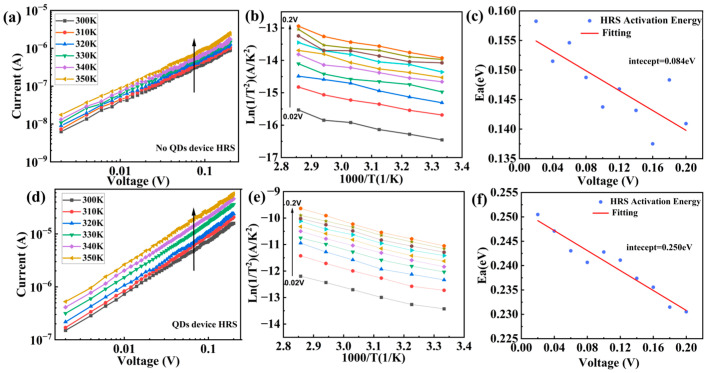
(**a**) The CV of HRS with temperature without QD memristors. (**b**) The ln(I/T2)−1000/T curve of HRS without QD memristors. (**c**) The activation energy fitting of HRS without QD memristors. (**d**) The CV of HRS with organic-QD hybrid memristors. (**e**) The ln(I/T2)−1000/T curve of HRS without QD memristors. (**f**) The activation energy fitting of HRS without QD memristors.

To further elucidate the conduction mechanism, we investigated the thermal response of the devices in the high-resistance state (HRS). As shown in Figure 5a,d, the HRS resistance of both device types decreased with increasing temperature, indicative of semiconducting transport behavior and consistent with a CFs rupture/formation model. The underlying energy barriers were quantified by replotting the HRS data in Arrhenius form ln(I/T^2^) vs. 1000/T, (Figure 5b,e). Linear fitting of these curves yielded the activation energy (Ea) for CF dissolution during the reset operation. A striking difference was observed: the PMMA-QD hybrid device exhibited a substantially higher activation energy of 0.25 eV, compared to only 0.084 eV for the QD-free control (Figure 5c,f). This threefold increase in Ea provides direct physical insight into the role of the QDs. It indicates that a greater energy input is required to rupture the Ag filaments in the hybrid device, unequivocally confirming that the QD-guided CFs possess a more robust and stable structure. This result directly correlates with the superior cycling endurance and switching uniformity, underscoring how the nanoscale field-confinement effect of the QDs mitigates random filament breakage and enhances overall device stability.

Collectively, these findings substantiate a model where the QDs, by intensifying the local electric field, direct the growth of Ag filaments along preferential pathways (Figure S11a–c). This guidance leads to the formation of fewer-defect, more stable CFs, in stark contrast to the random and fragile filament networks formed in the QD-free devices (Figure S12a–c), thereby explaining the superior performance and reliability of our hybrid memristor.

**Figure 6:**
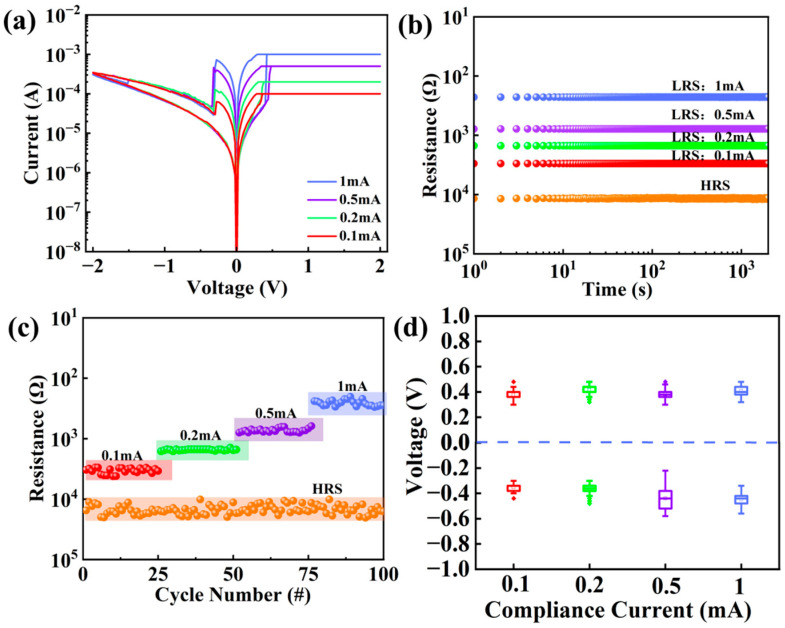
(**a**) I–V curves of the memristor under different compliance currents (0.1 mA, 0.2 mA, 0.5 mA, and 1 mA). (**b**) Retention characteristics of the memristor under different compliance currents (ICCs). (**c**) Statistical analysis of HRS/LRS under different ICCs. (**d**) Box plot statistics of set/reset threshold voltages under different ICCs.

The developed memristor demonstrates significant potential for high-density data storage through controllable multilevel resistance states [29,30,31]. By strategically modulating the compliance current (0.1, 0.2, 0.5, and 1 mA), four distinct and stable LRS were precisely programmed, as evidenced by the non-overlapping I–V curves in Figure 6a. Crucially, all LRS levels retained their integrity for over 2 × 10^3^ s at a read voltage of 0.1 V (Figure 6b), confirming robust non-volatility even under low-current operation. The clear separation of these four LRS levels from a single high-resistance state (HRS), statistically validated in Figure 6c, establishes a reliable five-state memory system capable of enhancing storage density beyond binary logic. Figure 6d presents a statistical analysis of the set/reset threshold voltages of the devices under different ICC conditions. Although the ICCs were significantly reduced, the set/reset threshold voltages exhibited no substantial variation, indicating that low ICCs had little effect on the resistive switching performance of the devices. At the same time, this multilevel adjustable resistance characteristic is also a necessary condition for simulating the properties of biological synapses. It lays the foundation for further exploration of its application in synaptic simulation. Additionally, the TIPS-pentacene:PMMA:CdSe/ZnS devices exhibit good stability without any significant degradation after 1, 3 and 7 days in an ambient environment (Figure S13a). The device performances show high consistency at different relative humidities (30%, 50%, and 80%) in Figure S13b and multiple temperatures (20, 50, 100 °C) in Figure S13c. The switching ratio remained stable, proving that this quantum dot/organic composite structure has excellent environmental thermal stability.

**Figure 7:**
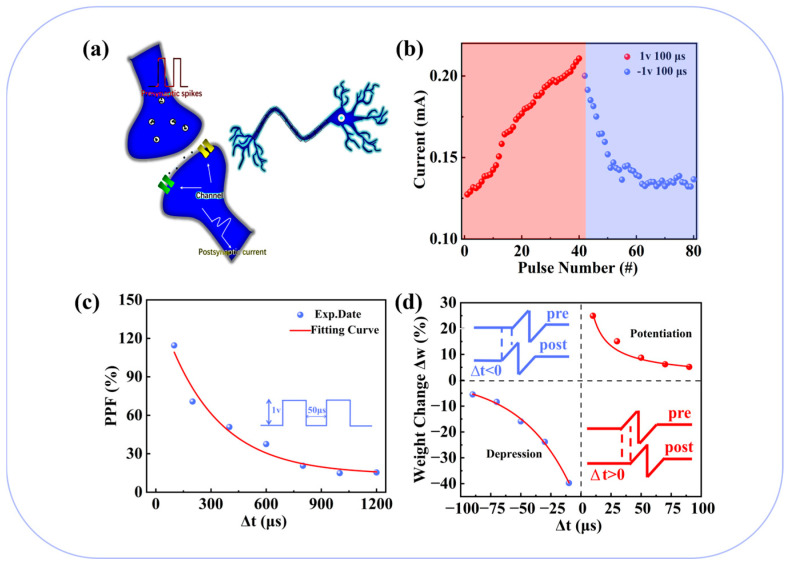
(**a**) Schematic illustration of the fundamental process of synaptic transmission in neurons. (**b**) Emulation of LTP and LTD behaviors of the device under multi-pulse modulation. (**c**) Effect of different pulse intervals on the PPF phenomenon. (**d**) STDP learning rule depicting synaptic weight changes in the memristor under various time delays (Δt).

Beyond memory, the device successfully emulates key neuromorphic functions [32,33,34]. We configured the Ag top electrode and FTO bottom electrode as pre- and post-synaptic terminals, respectively, where Ag^+^ ion migration under electrical stimulation mimics neurotransmitter release Figure 7a. The device exhibits continuous conductance modulation in response to input spikes, implementing fundamental synaptic plasticity. As shown in Figure 7b, the synaptic weight, represented by the device current, demonstrates progressive long-term potentiation (LTP) under 40 positive pulses (+1 V, 100 μs) and long-term depression (LTD) under 40 negative pulses (−1 V, 100 μs). To further demonstrate the neuromorphic capabilities of our device, we successfully implemented paired-pulse facilitation (PPF), a fundamental short-term plasticity mechanism in biological synapses where residual calcium from an initial stimulus enhances neurotransmitter release in response to a subsequent spike. This biological phenomenon was replicated by applying consecutive voltage pulses (1 V, 50 μs) with varying temporal intervals (Δt) to our memristor. As illustrated in Figure 5c, the second pulse consistently evoked a higher current response (A2) compared to the first (A1), demonstrating clear facilitation. The PPF index was quantified as follows:

(2) $${\mathrm{PPF}}_{\mathrm{index}\;}=\;\frac{A_{2}-A_{1}}{A_{1}}\;\times \;100\%$$

This index exhibited characteristic exponential decay with increasing Δt, accurately described by the following:

(3) $$Y\;=\;C\;+\;Ae^{-t/\tau }$$

Notably, the PPF index reached 124% at Δt = 100 μs, progressively decaying from 118% to 13.4% as Δt increased. This temporal dependence of the facilitation effect confirms the device’s inherent short-term memory retention capability, where the retention strength decreases with increasing pulse interval, a behavior that closely mirrors the calcium dynamics underlying PPF in biological synapses. The successful emulation of this fundamental neural mechanism underscores the potential of our PMMA-QD hybrid memristor for implementing complex temporal processing in neuromorphic computing systems.

In addition, the device successfully emulates spike-timing-dependent plasticity (STDP), a fundamental unsupervised learning rule in biological neural systems where synaptic strength is modulated by the precise temporal sequence of pre- and postsynaptic spikes. To implement this mechanism, we applied precisely controlled alternating triangular pulses (±1 V) to the Ag (presynaptic) and FTO (postsynaptic) electrodes while systematically varying their temporal separation (Δt). The resulting synaptic weight modification follows the quantitative relationship below:

(4) $$\Delta w\;=\frac{G_{1}-G_{0}}{G_{0}}\;\times \;100\%$$

Experimental results reveal that the synaptic efficacy obeys a double-exponential dependence on spike timing:

(5) $$\Delta w\;=\;\lambda \exp\left(-K\left|\Delta t\right|\right)$$

The measured STDP window exhibits the characteristic biological asymmetry, achieving a maximum potentiation of +24.94% when presynaptic activation precedes postsynaptic firing (Δt = +10 μs), and a maximum depression of −39.76% for the reverse temporal sequence (Δt = −10 μs), as shown in Figure 7d. This result definitively establishes the influence of temporal signal ordering on the synaptic behavior of our device.

Comparative analysis with existing technologies (Table 1) reveals that our memristor outperforms other reported devices, marked by its ultralow operating voltages, robust cycling endurance exceeding 1200 cycles, and stable retention over 10^4^ s. This superior performance underpins its unique potential for implementing high-density memory and complex neuromorphic functions. Further improving film uniformity during spin-coating is expected to reduce device variability and enhance array-level stability for future high-density neuromorphic applications.

## 4. Conclusions

In summary, we have developed a solution-processed ternary hybrid organic memristor based on TIPS-pentacene, PMMA, and CdSe/ZnS quantum dots, and demonstrated a dual-engineering strategy for simultaneously regulating semiconductor crystallization and conductive filament formation. PMMA serves as a crystallization template that improves the continuity and molecular ordering of the TIPS-pentacene film, whereas CdSe/ZnS quantum dots function as nanoscale electric-field modulators that guide and confine Ag filament growth. Owing to this synergistic design, the optimized device exhibits ultralow and highly uniform switching voltages (0.473/−0.430 V), low resistance-state fluctuation, retention exceeding 10^4^ s, and endurance over 1200 cycles. The device further supports multilevel memory operation and emulates key synaptic plasticity functions, including LTP/LTD, PPF, and STDP. These results establish a viable materials-engineering route toward reliable, low-power organic memristors and provide useful design principles for future neuromorphic and high-density nonvolatile memory systems.

## Figures and Tables

**Table 1:** Comparison of PMMA-QD hybrid memristors with CdSe QD-based memristors and other organic memristors.

Memristor	Threshold	Retention	Endurance	Multilevel Storage	Synaptic Activity	Refs.
Al/CdSe/ZnS/PMMA/ITO	2.5/−2	10^4^	500	—	—	[35]
Ag/gelatin: CdSe/ZnS/ITO	0.75/−2.37	10^4^	100	√	—	[36]
Al/ET-COF/ITO	2.55/−0.65	10^4^	100	√	√	[37]
Al/MoS_2_-PDA-tBu_4_PcGaCl/ITO	0.7/−3	10^4^	200	—	√	[32]
Au/DTA-me-DTPZ:CN-T2T/ITO	2/−5.5	—	300	—	√	[38]
Al/COF-NUC-1/ITO	1.82/−0.68	>10^4^	200	√	—	[39]
Al/PEDOT:PSS/pentacene/ITO	1/−1	—	—	—	√	[40]
Ag/PMMA/QDs/TIPS-pentacene/FTO	0.47/−0.43	10^4^	>10^3^	√	√	this work

## Data Availability

The original contributions presented in this study are included in the article/Supplementary Materials. Further inquiries can be directed to the corresponding authors.

## Supplementary Materials

The following supporting information can be downloaded at https://www.mdpi.com/article/10.3390/ma19143029/s1: Figure S1: Spin-coating process and fabrication procedure of organic-quantum dot hybrid memristor; Figure S2: (a) Optical microscope image of a TIPS-pentacene film spin-coating on a FTO substrate from a 2 mg/mL solution. (b) Optical microscope image of a film from a 6 mg/mL solution. (c) Optical microscope image of a film from a 10 mg/mL solution. (d) I–V curve of an Ag/TIPS-pentacene/FTO memristor prepared with a 2 mg/mL solution. (e) I–V curve of a memristor prepared with a 6 mg/mL solution. (f) I–V curve of a memristor prepared with a 10 mg/mL solution; Figure S3: High-resolution SEM image of the as-fabricated CdSe/ZnS QDs film; Figure S4: (a) Optical microscope image of a blend film with a PMMA concentration of 6 mg/mL. (b) Optical microscope image of a blend film with a PMMA concentration of 10 mg/mL. (c) I–V curves of a memristor fabricated from a solution with 6 mg/mL PMMA. (d) I–V curves of a memristor fabricated from a solution with 10 mg/mL PMMA; Figure S5: (a) EDS elemental mapping of the TIPS-pentacene:PMMA:CdSe/ZnS composite film. (b–f) C, O, Si, Cd, Se, Zn, and S maps confirming homogeneous dispersion throughout the polymer matrix; Figure S6: UV-absorption spectra of CdSe/ZnS QDs, Tips-pentacene and the mixed solution; Figure S7: SEM cross-sectional image of the memristor; Figure S8: Cumulative probability distribution of HRS/LRS for memristors with different proportions of CdSe/ZnS QDs: (a) No QDs, (b) QDs:TIPS-pentacene is 1:2, (c) QDs:TIPS-pentacene is 2:1; Figure S9: (a–h) The I–V cycling test curves of ten different devices with organic-quantum dot hybrid memristor; Figure S10: Retention of CdSe/ZnS QDs memristors with different proportions introduced: (a) No QDs, (b) QDs:TIPS-pentacene is 1:2, (c) QDs:TIPS-pentacene is 2:1; Figure S11: Model of QDs induced directed filamentation: (a) Initial state, (b) conductive state, (c) non-conductive state; Figure S12: Model of undirected filamentation without QDs: (a) Initial state, (b) conductive state, (c) non-conductive state; Figure S13: Stability tests of the QDs:TIPS-pentacene memristor, (a) I–V curves of the memristor after 1 day, 3 days and 7 days, (b) I–V curves of devices exposed to 30%, 50% and 80% relative humidity, (c) I–V curves of the device at different temperatures.
