# Supplementary material for: Stable Low-Voltage Organic Memristors Enabled by Templated Crystallization and Quantum-Dot-Regulated Filament Formation
Source: Materials (Basel). 2026 Jul 14;19(14):3029. doi: 10.3390/ma19143029 (PMC13413562; doi:10.3390/ma19143029)
Supplement: Supplementary file 1 [file materials-19-03029-s001.zip › materials-4406174-supplementary.pdf]

Supporting Information

Stable Low-Voltage Organic Memristors Enabled by Templated Crystallization and Quantum-Dot-Regulated Filament Formation

Qi Lei [a,‡], Yonghua Tu [a,‡], Zilong Yan [a], Junqing Wei [a,*], Boning Han [b],

Haiwei Zhang [a], Yangyang Xie [a,*], Kailiang Zhang [a]

[a] Tianjin Key Laboratory of Film Electronic and Communication Devices, School of Integrated Circuit Science and Engineering, Tianjin University of Technology, Tianjin 300384, China.

[b] Tianjin Key Laboratory for Photoelectric Materials & Devices, School of Materials Science and Engineering Tianjin University of Technology, Tianjin 300384, China.

‡ These authors contribute equally to this work.

***Corresponding authors.**

E-mail address: xyy0905@email.tjut.edu.cn (Yangyang Xie)

weijunqing0907@163.com (Junqing Wei)

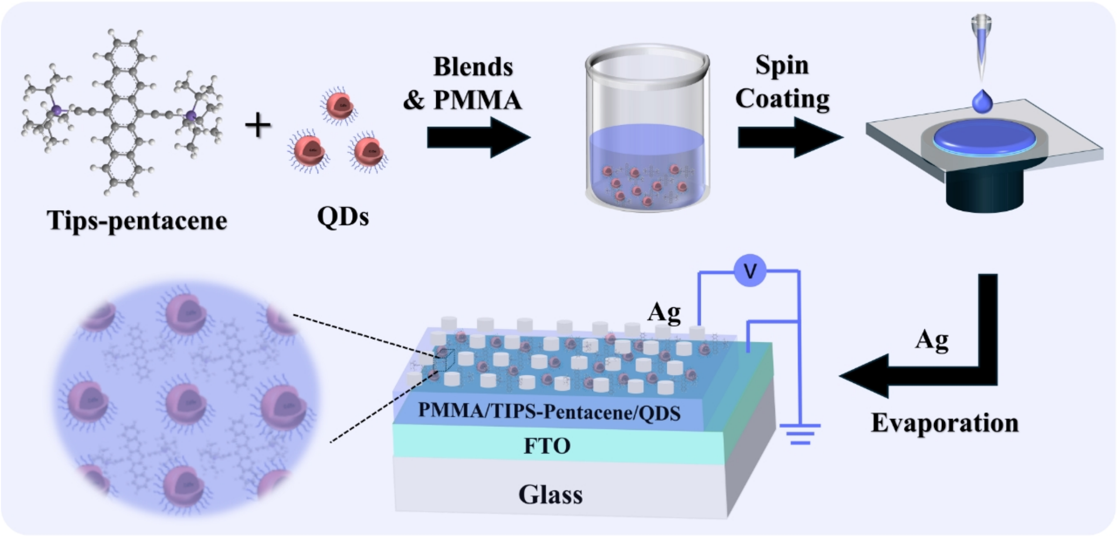

Figure S1. Spin-coating process and fabrication procedure of organic-quantum dot hybrid memristor.

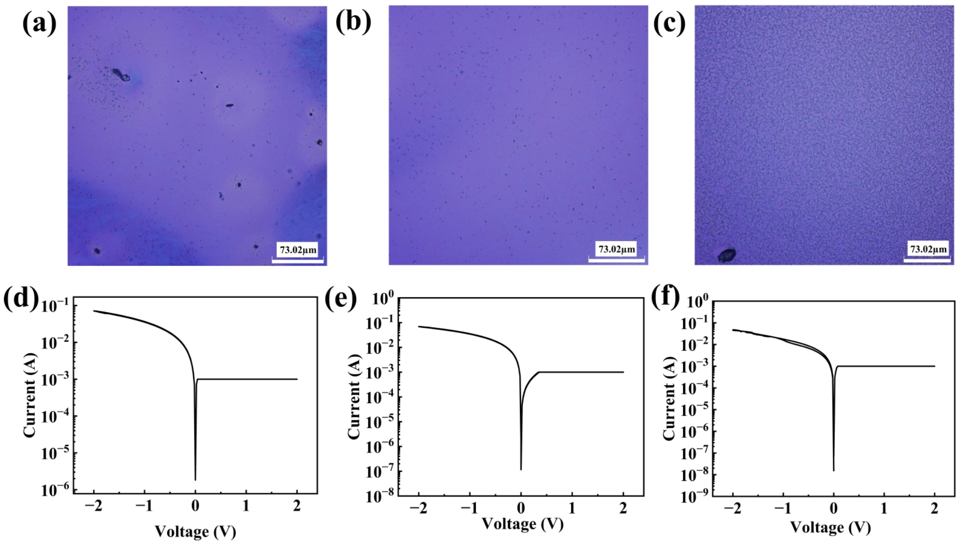

Figure S2. (a) Optical microscope image of a TIPS-pentacene film spin-coating on a FTO substrate from a 2 mg/mL solution. (b) Optical microscope image of a film from a 6 mg/mL solution. (c) Optical microscope image of a film from a 10 mg/mL solution. (d) I-V curve of an Ag/TIPS-pentacene/FTO memristor prepared with a 2 mg/mL solution. (e) I-V curve of a memristor prepared with a 6 mg/mL solution.(f) I-V curve of a memristor prepared with a 10 mg/mL solution.

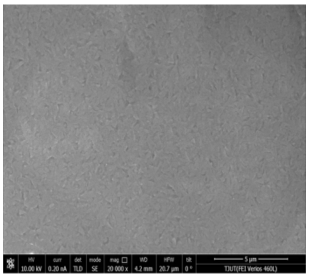

Figure S3. High-resolution SEM image of the as-fabricated CdSe/ZnS QDs film.

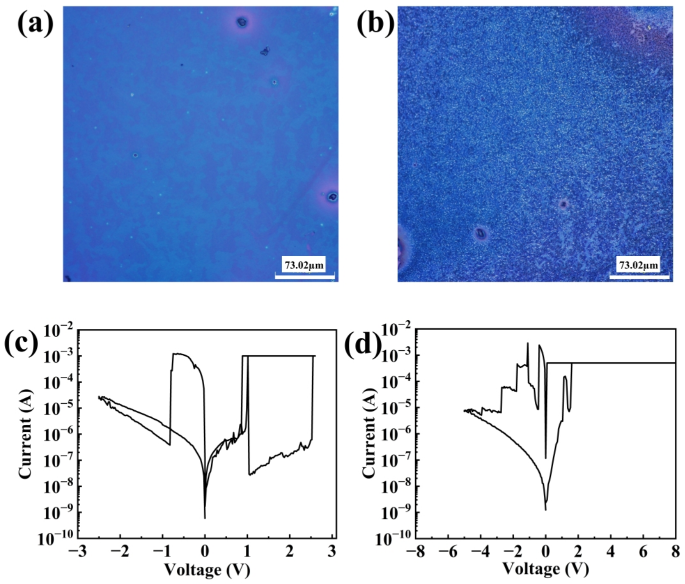

Figure S4. (a) Optical microscope image of a blend film with a PMMA concentration of 6 mg/mL. (b) Optical microscope image of a blend film with a PMMA concentration of 10 mg/mL. (c) I-V curves of a memristor fabricated from a solution with 6 mg/mL PMMA. (d) I-V curves of a memristor fabricated from a solution with 10 mg/mL PMMA.

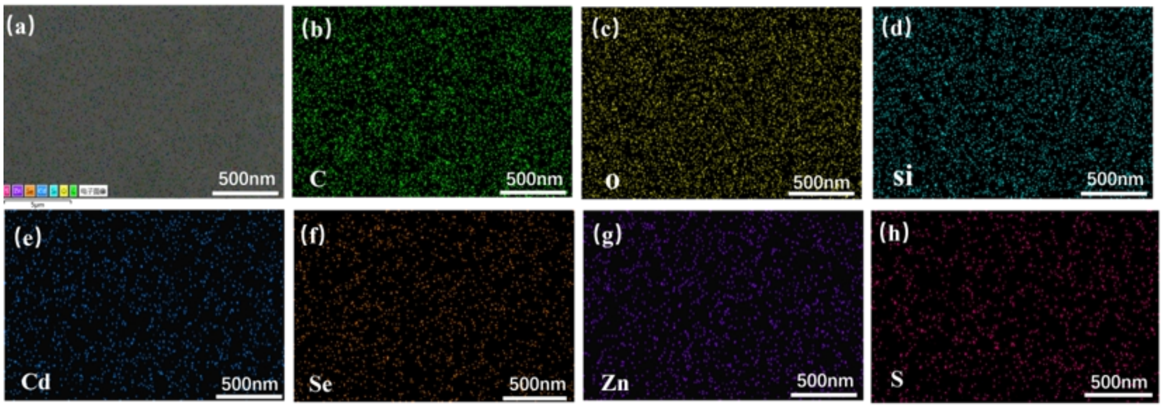

Figure S5. (a) EDS elemental mapping of the TIPS-pentacene:PMMA:CdSe/ZnS composite film. (b-f) C, O, Si, Cd, Se, Zn, and S maps confirming homogeneous dispersion throughout the polymer matrix.

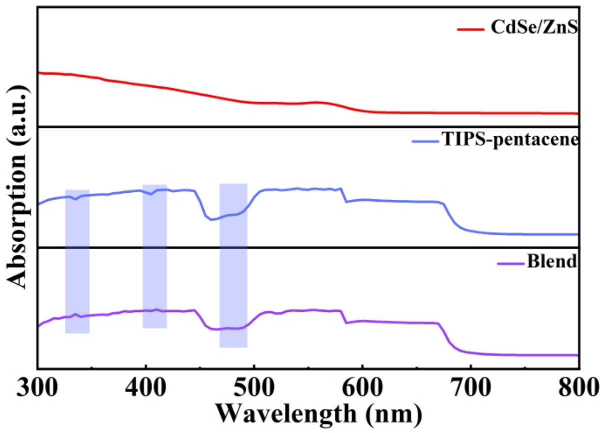

Figure. S6. UV-absorption spectra of CdSe/ZnS QDs,Tips-pentacene and the mixed solution.

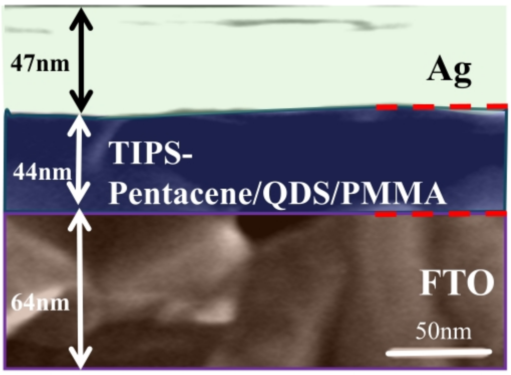

Figure S7. SEM cross-sectional image of the memristor.

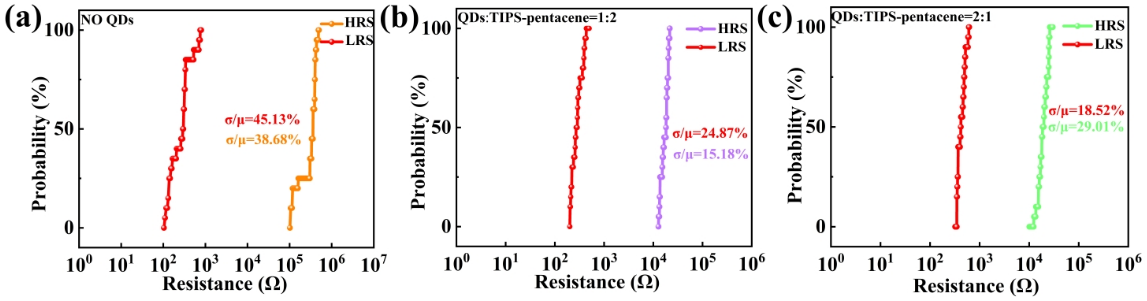

Figure S8. Cumulative probability distribution of HRS/LRS for memristors with different proportions of CdSe/ZnS QDs: (a) No QDs, (b) QDs:TIPS-pentacene is 1:2, (c) QDs:TIPS-pentacene is 2:1.

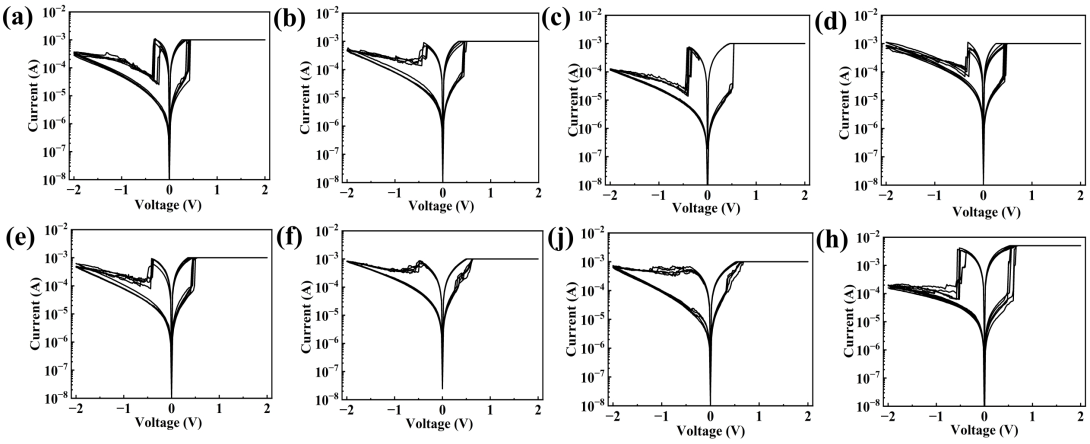

Figure S9. (a-h) The I-V cycling test curves of ten different devices with organic-quantum dot hybrid memristor.

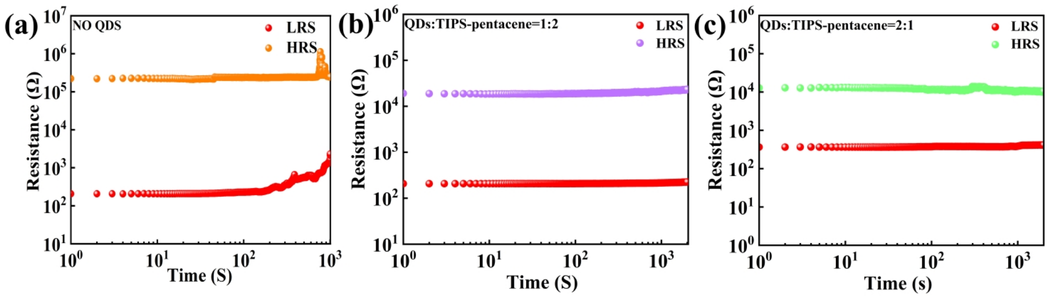

Figure S10. Retention of CdSe/ZnS QDs memristors with different proportions introduced:(a) No QDs, (b) QDs:TIPS-pentacene is 1:2, (c) QDs:TIPS-pentacene is 2:1.

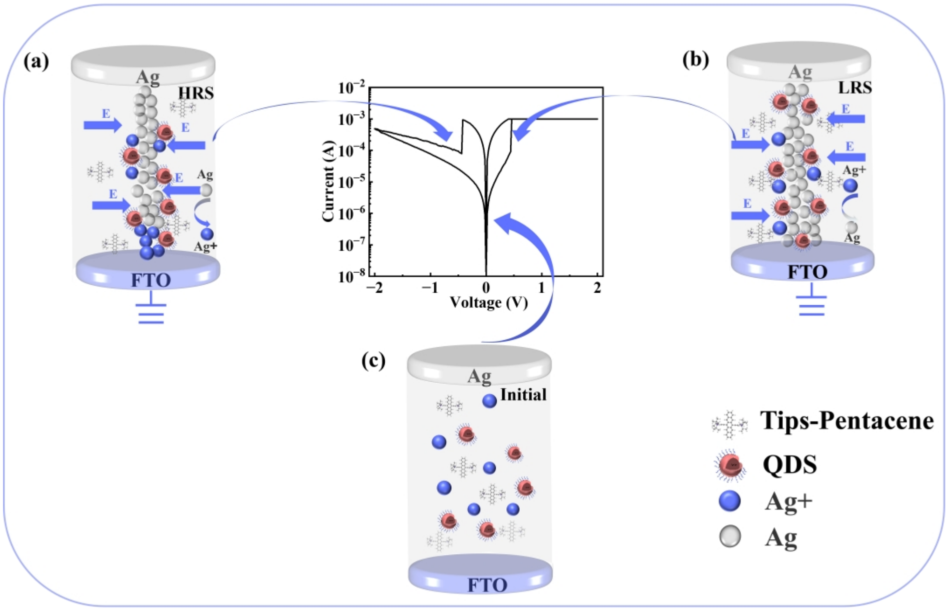

Figure S11. Model of QDs induced directed filamentation: (a) Initial state, (b) conductive state, (c) non-conductive state.

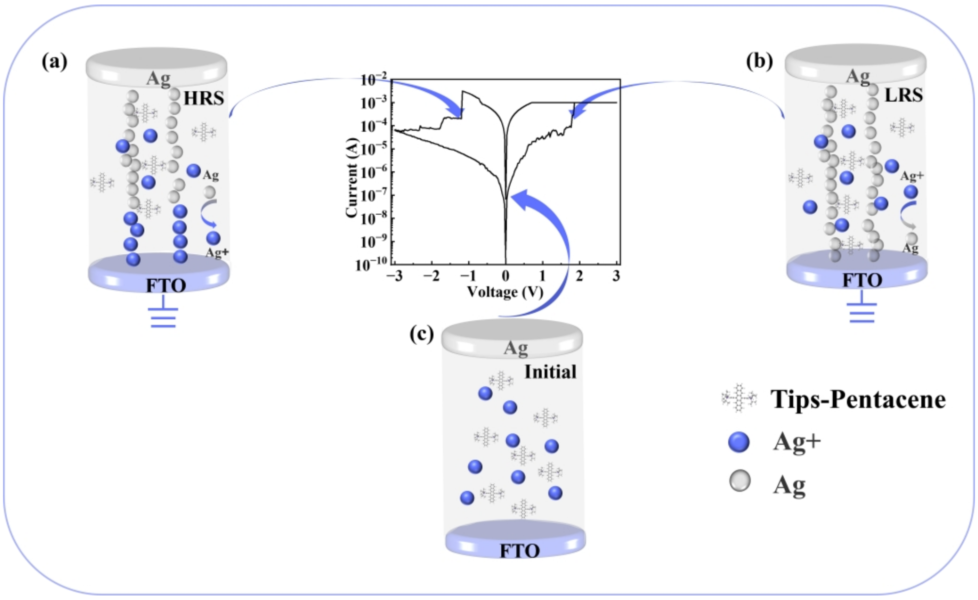

Figure S12. Model of undirected filamentation without QDs: (a) Initial state, (b) conductive state, (c) non-conductive state.

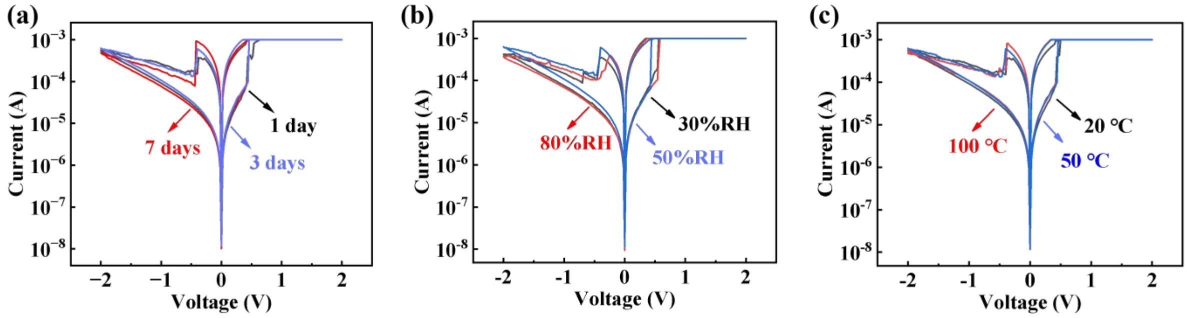

Figure S13. Stability tests of the QDs:TIPS-pentacene memristor, (a) I-V curves of the memristor after 1 day,3 days and 7 days, (b) I-V curves of devices exposed to 30%, 50% and 80% relative humidity, (c) I-V curves of the device at different temperatures.
